# Impact of Storage at −80°C on Encapsulated Liver Spheroids After Liquid Nitrogen Storage

**DOI:** 10.1089/biores.2016.0017

**Published:** 2016-06-01

**Authors:** Peter Kilbride, Jordi Gonzalez-Molina, Natasha Maurmann, Joana Mendonça da Silva, Stephanie Gibbons, Clare Selden, Barry Fuller, John Morris

**Affiliations:** ^1^ Asymptote Ltd., St. John's Innovation Centre, Cambridge, United Kingdom.; ^2^ UCL Institute for Liver and Digestive Health, Royal Free Hospital Campus, London, United Kingdom.; ^3^ UCL Department of Surgery, Royal Free Hospital Campus, London, United Kingdom.

**Keywords:** alginate encapsulation, bioartificial liver device, cryopreservation, HEPG2 cells, warming rates

## Abstract

For many bioengineered tissues to have practical clinical application, cryopreservation for use on demand is essential. This study examined different thermal histories on warming and short holding periods at different subzero temperatures on subsequent functional recoveries of alginate encapsulated liver spheroids (ELS) for use in a bioartificial liver device. This mimicked transport at liquid nitrogen (−196°C) or dry ice (∼−80°C) temperatures. Holding at −80°C on warming after −196°C storage resulted in ELS expressing significant (*p* < 0.001) damage compared with direct thaw from liquid nitrogen, with viable cell number falling from 74.0 ± 8.4 million viable cells/mL without −80°C storage to 1.9 ± 0.6 million viable cells/mL 72 h post-thaw after 8 days storage at −80°C. Even 1 day at −80°C after −196°C storage resulted in lower viability (down 21% 24 h post-thaw), viable cell count (down 29% 24 h post-thaw), glucose, and alpha-1-fetoprotein production (reduced by 59% and 95% 24 h from 1 day post-thaw, respectively). Storage at −80°C was determined to be harmful only during the warming cycle. Chemical measurements of the alginate component of ELS were unchanged by cryogenic exposure in either condition.

## Introduction

Successful and reliable transportation and distribution of bioengineered products from point of manufacture to point of care are essential for clinical application. Many biological constructs, such as the 2-L bioartificial liver (BAL) device being developed by the authors,^1^ require storage at cryogenic temperatures (below −150°C) for effective delivery and treatment of acute liver failure. At cryogenic temperatures, samples can be stored indefinitely^2,3^ before thawing on demand. Most complicated bioengineered products take several weeks or months to produce and so just in time manufacture is not logistically or economically feasible for patient treatment.

Methods of transportation have wide consequences for all cryopreserved constructs. Transportation of biological agents—both bioengineered constructs and direct specimens such as spermatozoa, tissue samples, and embryos—is an involved issue, but largely can be categorized into two groups as follows: transport at liquid or vapor phase nitrogen temperatures using commercial dry shippers typically between −196°C and −150°C, or transport at dry ice temperatures (∼−80°C).^4–7^

A dry shipper is a liquid nitrogen storage container, pre-charged with cryogen, into which samples can be placed and transported. As the liquid nitrogen is adsorbed into zeolites, there is little risk of liquid nitrogen spillage. Liquid nitrogen is categorized as a dangerous product during shipping however, and so transport can be relatively expensive. Dry shippers have a finite transportation window before uncontrolled warming occurs since they cannot easily be “topped up” during transport. Storage and transport with liquid nitrogen also comes with increased risk of biomass contamination as liquid nitrogen is routinely nonsterile, although this risk may be low for the transport stage.^8–11^

There are many studies where different tissue types have been transported successfully at liquid nitrogen temperatures (LN_2_).^4–6,12,13^ In addition, liquid nitrogen temperatures are essential for vitrified samples, as transport at −80°C (above the glass transition temperature for many common cryoprotectants) would normally result in the samples devitrifying extracellularly on warming, inducing significant biological injury.

Transport at dry ice temperatures is also used widely and is cheaper and easier in many practical senses.^5^ The setup for short transport is generally straightforward, and can be as simple as a polystyrene box filled with dry ice. This allows for the transport of large and irregularly shaped samples easily and does not require the receiver to have liquid nitrogen handling infrastructure. In addition, −80°C freezers are widely available commercially and found routinely in the majority of potential treatment centers and so, samples transported on dry ice can be placed in a suitable freezer on arrival without temperature disruption for short or longer term storage. This can provide flexibility for the end user if products cannot be used on the day of delivery or operations need to be rescheduled.

In addressing these topics, it has previously been demonstrated by our group that encapsulated liver spheroids (ELS) can be stored for at least 1 year at liquid nitrogen temperatures without significant loss of function, while storage at −80°C for periods a month and over shows damage to the cells; this mirrors results observed with many other cell types, which require liquid nitrogen temperatures for successful long term storage.^10,14^

This study examined, from a cryobiological viewpoint, which of the two temperatures of likely transportation in the cryogenic state (−80°C or −196°C) is optimal for the ELS comprising the BAL device.

## Materials and Methods

### Pre-cryopreservation culture methods

#### Cell culture and encapsulation

An immortalized hepatocyte cell line (HepG2) was cultured in static flasks (triple layer) for 2 weeks and passaged twice to produce required cell numbers. These cells were then added to alginate in aqueous solution, with a final concentration of 2 × 10^6^ cells/mL and 1% alginate (w/v). 0.75% glass beads (w/v) were added to the alginate/cell mix to act as a buoyancy regulator during fluidization.

This alginate/cell/glass mix was passed through a geniaLab JetCutter system to produce alginate ELS, which were cross-linked with 0.204 M aqueous calcium. These alginate beads had a diameter of 500 μm and clusters of spheroids developed in this volume during culture. These were then added to a fluidized bed bioreactor. This culture method has previously been described in detail.^1,14,15^

#### Fluidized bed culture

Approximately, 2 L of the ELS biomass was added to a fluidized bed bioreactor and cultured using alpha-modified Eagle's medium supplemented with 10% (v/v) human plasma (National Blood Transfusion Service). After 12 days' culture, the biomass had achieved a sufficient cell density, at which point samples were removed for cryopreservation.^1^

### Cryopreservation protocols

#### Cooling protocol

A cryoprotectant mix was prepared and stored at 4°C, consisting of 24% v/v dimethyl sulfoxide (DMSO; Sigma), in University of Wisconsin Solution (UW; Organ Recovery Systems). One milliliter of this solution was added to 1 mL of ELS in 2 mL cryovials, resulting in a final concentration of 12% DMSO, 50% ELS, and 38% UW v/v. 0.1% cholesterol w/v was included as an ice nucleator.^15,16^

These samples were stored on ice and added to an EF600 controlled rate freezer. The EF600 was programmed to cool from 4°C to −80°C at −0.3°C/min. Upon completion of the cooling profile, samples were either transferred to liquid nitrogen or stored in a −80°C freezer (Sanyo), depending on the condition being tested.

### Thawing protocols

#### Positive control

In these experiments, the positive controls were not unfrozen samples, rather it was samples cryopreserved, stored in, and warmed directly from liquid nitrogen—the current optimized protocol. These did not include any −80°C hold step, the condition under review in this set of experiments.

These samples were removed from liquid nitrogen storage after 14 days and took 330 sec to thaw in a 37°C water bath, before the cryoprotectant was removed and samples returned to culture. This rapid warm from liquid nitrogen is the current optimized method.^15,17^

#### Samples stored at −80°C after liquid nitrogen storage

To investigate the effects of storage at −80°C after liquid nitrogen storage, mimicking warmer transportation temperatures, samples were placed into an insulated box at liquid nitrogen temperatures; this box was then placed into a −80°C freezer, with the samples equilibrating to −80°C after ∼1 h.

Samples were thawed rapidly in a 37°C water bath after storage of 1, 4, or 8 days at −80°C. The thawing process took 200 sec from −80°C. Upon thawing, the cryoprotectant was diluted out and the samples returned to culture.^14,15^

The total time samples were cryopreserved for 14 days.

#### Samples stored only at −80°C

To explore the impact of −80°C storage over the shorter term where the sample was never exposed to liquid nitrogen temperatures even during the cooling step, samples were removed from the EF600 after the cooling profile had reached −80°C and stored in a −80°C freezer. Samples were then removed after 1, 4, and 8 days, thawed in 200 sec in a 37°C water bath, and returned to culture.

#### Removal of cryoprotectant

Upon sample thaw, the ELS and freezing mix were emptied from the cryovial into 50 mL centrifuge tubes. To the mix, 1 mL of Hanks' buffered saline solution (HBSS) chilled to 4°C was added. At 1 min intervals, 2, 4, 8, 16, and 16 mL were added to the mix that was gently stirred between steps. The solution was allowed to equilibrate for 5 min, after which the supernatant was removed and the ELS added to a Rotary Cell Culture System (RCCS; Synthecon) culture containing warmed culture medium.^18^

#### Post-thaw culture

The samples were added to the RCCS, which was operated at 37°C in a 5% CO_2_ humidified incubator and rotated at a rate of 10 rotations/min. The RCCS produced a microgravity environment mimicking the fluidized bed bioreactor.^18^

Samples were removed with a pipette at 24, 48, and 72 h culture intervals for post-thaw assessments to be carried out.

### Post-thaw assessments

#### Viability assays

A cell membrane viability assay was carried out using propidium iodide (PI) and fluorescein diacetate (FDA) staining as previously described. PI dyes the nucleus of cells with a permeated membrane, while FDA only stains metabolically active cells. Measurement and quantification of fluorescence were carried out using a Nikon Imaging Suite software with a validated macro.^1,15^

#### Cell count techniques

To determine cell counts, liver cells were liberated from the alginate beads with a 16 mM EDTA solution. The cell spheroids were disaggregated and then quantified using a NucleoCounter device. Cell counts and viabilities were then merged to give a viable cell number.^1,15^

#### Enzyme-linked immunosorbent assay

Protocol ELS produce alpha-1-fetoprotein (AFP), the level of which was measured with sandwich enzyme-linked immunosorbent assays (ELISAs) with primary antibody sourced from Abcam (Cat. No. ab10071) and Abcam supplying the secondary, HRF-linked antibody (Cat. No. ab10072); this was quantified with a standard curve of human serum-derived AFP (AppliChem; Cat. No. A6935). Data were normalized to per mL ELS.

#### Glucose measurements

Glucose levels in the culture medium were established using an Analox GM7 through enzyme-linked oxygen rate measurements. Medium samples were removed from culture, frozen at −20°C. On measurement, the samples were thawed and glucose measured, with calibration and washes carried out using a glucose standard (Analox) and reagent (Analox GMRD-002A).

#### Fourier transform infrared spectroscopy

To determine whether physical changes in the alginate could be a source of cell death during the cryopreservation, Fourier transform infrared (FTIR) spectroscopy was carried out on alginate beads pre- and post-thaw. Whole alginate beads were washed four times with a solution containing 1.57 mM CaCl_2_ 0.155 M NaCl at pH 7.4, and 50–100 μL of beads were used for spectra recordings per condition. Mid-IR spectra were recorded with a Bruker IFS/66S FTIR spectrophotometer fitted with a liquid nitrogen-cooled MCT-B detector at 4 cm^−1^ resolution. All cited frequencies have an accuracy of ±1 cm^−1^. FTIR spectra were obtained within the range between 4000 and 850 cm^−1^ during 250 scans. Spectra of alginate beads and CaCl_2_ solutions were recorded separately for subsequent subtraction of the CaCl_2_ solutions from each bead sample's spectrum.

### Statistics

In all cases, significance was determined by use of a Student's *t*-test. Significance was observed at *p* < 0.05, *p* < 0.01, and *p* < 0.001—the level stated with each experiment.

## Results

### Viability and viable cell number

**FIG. 1.**
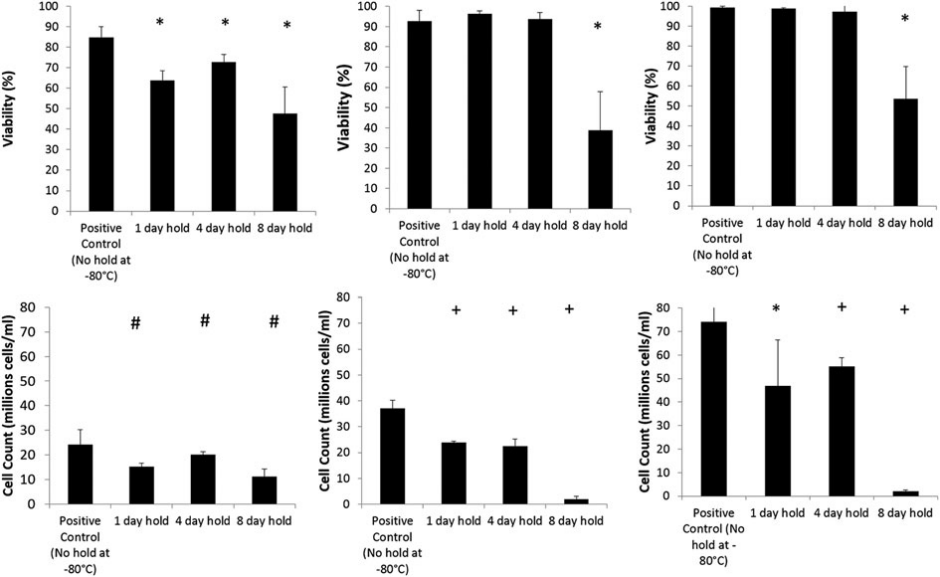
Viability (top) and viable cell number (bottom) of samples cooled to −196°C, warmed to −80°C, and held for stated periods of time. The total time from freezing to thaw was 14 days. Viability and cell count measurements were made at 24 h (left), 48 h (center), and 72 h (right) post-thaw. Significance from a control that was thawed without stopping at −80°C is denoted by ^#^*p* < 0.05, *p < 0.01, and ^+^*p* < 0.001. *n* = 5 ± SD.

Samples held at −80°C (1, 4, or 8 days), following storage at −196°C, have significantly worse viability on thawing compared with a sample thawed directly from −196°C (Fig. 1). This difference is at maximum at 24 h post-thaw. No significant difference was measured at the 48 and 72 h post-thaw culture time points for 1 and 4 day holds compared with a direct thaw. In samples held for either 1 or 4 days at −80°C, samples held at −80°C for 8 days after liquid nitrogen storage did not recover viability by 72 h post-thaw.

Viable cell numbers, which take into account recovered cell counts (i.e., not those destroyed and washed out from the system after cryopreservation) and viabilities, show that the controls (thawed directly from −196°C) were significantly better than those held at any time points at −80°C. While those held for 1 and 4 days at −80°C show recovery by 48 h post-thaw, those held for 8 days did not show any recovery even by 72 h post-thaw.

**FIG. 2.**
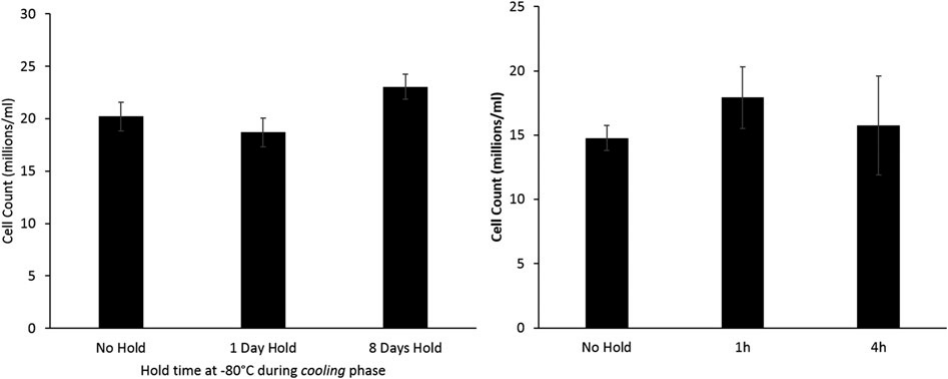
Left: comparing viable cell numbers of samples stored at −80°C on cooling to liquid nitrogen storage, but experienced a direct thaw from liquid nitrogen on warming—no significant difference is observed between any of the sets 24 h post thaw indicating that −80°C storage is not harmful during the cooling profile. Right: a separate set of samples that was cooled directly to liquid nitrogen temperatures, but stored for either 1 or 4 h at −80°C on warming and compared with samples cooled and thawed without a stop. No difference was observed between any of these sets. *n* = 5 ± SD.

However, Figure 2 (left) shows that for samples stored at only −80°C, there was no significant difference between storage times of 1–8 days.

To establish if the interruption to the warming cycle at −80°C was a damaging factor to cryopreservation *per se*, a separate set of samples stored at −196°C was warmed to −80°C and held for a period of either 1 or 4 h. No significant difference in viable cell number was observed between these samples and a set that was thawed directly from −196°C (Fig. 2; right).

**FIG. 3.**
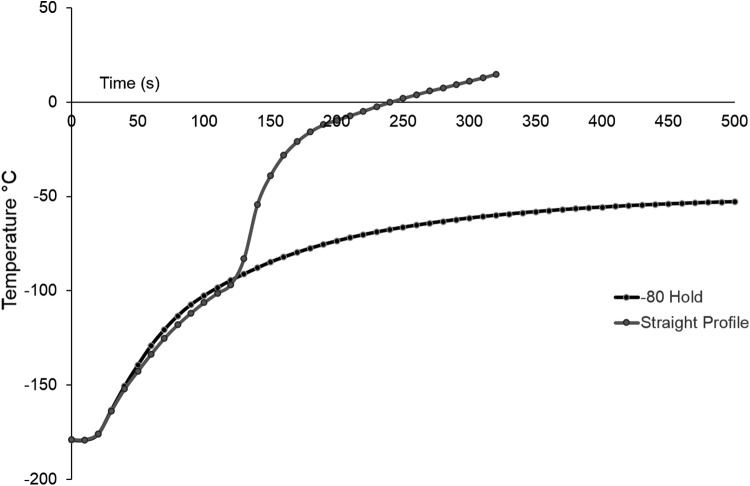
Comparing the difference in warming rates between samples thawed directly from liquid nitrogen (straight profile), with ones with a hold at −80°C. No significant difference was observed between either set below −80°C. The warming profile consisted of warming in air to −80°C, after which direct thaw samples were placed into a 37°C water bath, and samples held at −80°C were placed into a −80°C freezer before thaw in a 37°C water bath. Data are mean of five measurements per set.

Figure 3 shows the differences in warming profiles between samples warmed directly from liquid nitrogen to thaw compared with those experiencing a hold at −80°C. No significant difference is seen in warming between −196°C and the hold commencing at −80°C, excluding warming rate differences between these two conditions as a cause for differences in post-thaw outcome.

### Glucose consumption

**FIG. 4.**
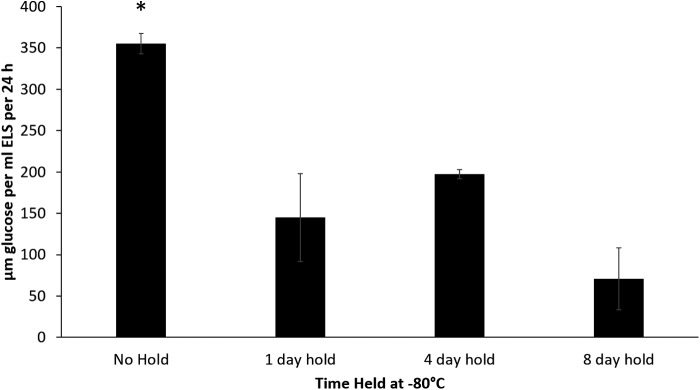
Glucose consumption levels measured in culture medium 2 days post-thaw of samples experiencing different lengths of storage at −80°C. The samples with no hold consumed significantly more glucose than those experiencing holds of any duration (indicated by *p < 0.001). No significant difference was seen between any of the other sets, *n* = 5 ± SD.

In the 48 h post-thaw, glucose consumption between sets thawed directly from −196°C (positive control) or stored at either −80°C for 1, 4, or 8 days on thaw was 355 ± 12, 144 ± 53, 197 ± 6, and 71 ± 37 μM per mL ELS per 24 h, respectively. Any storage time at −80°C post-LN_2_ resulted in significantly lower glucose consumption, as is shown in Figure 4.

### Protein synthesis by AFP ELISAs

**FIG. 5.**
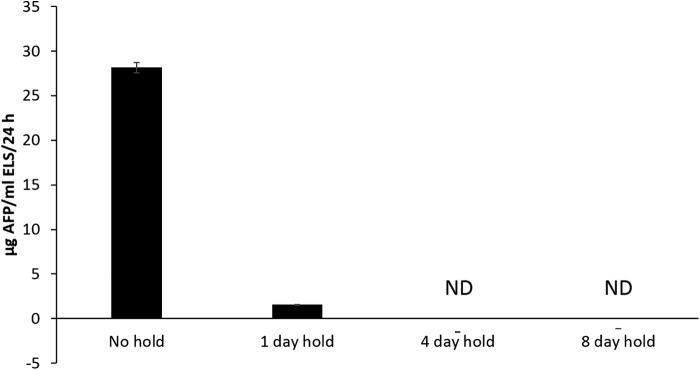
Alpha-1-fetoprotein (AFP) production per mL ELS per 24 h of samples experiencing different lengths of hold at −80°C after −196°C storage. These measurements were taken at 2 days post-thaw; the samples with no hold at −80°C had a significantly (*p* < 0.001) better AFP production over all other sets. With the exception of samples held for 1 day at −80°C, all samples that experienced a hold at −80°C on warming had negligible AFP production, with ND indicating none detected. *n* = 5 ± SD. ELS, encapsulated liver spheroids.

As can be seen from Figure 5, 2 days post-thaw, ELS that had been stored at −196°C and were thawed directly exhibited an AFP production of 28.1 ± 2.7 μg of AFP per mL ELS per 24 h.

The samples held for 1 day at −80°C after LN_2_ storage exhibited significantly reduced AFP production (*p* < 0.001) with a production of 1.6 ± 0.1 μg of AFP per mL ELS per 24 h.

Sets experiencing a 4- or 8-day hold at −80°C had negligible AFP production. As AFP is unstable *in vitro*, very low values may indicate residual AFP in the culture medium as degrading, with no new AFP production detected.^19^

While the HepG2 cell line used in ELS produces clinically relevant proteins such as albumin, fibrinogen, and alpha-1-antitrypsin, these are all present normally in large quantities in human blood plasma. As the culture media contained 10% human blood plasma, levels of these proteins cannot easily be observed above background levels.^1,15^ AFP is not present in normal human plasma and is in this study used as a surrogate for clinically relevant protein synthetic activity.^1^

### Chemical assessment of alginate in ELS by FTIR

**FIG. 6.**
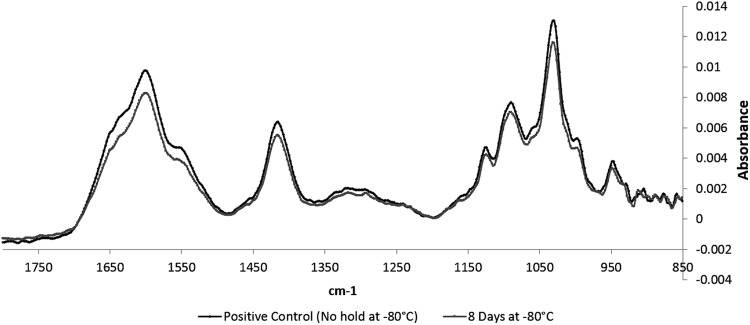
Absorbance measured between samples experiencing no hold at −80°C and samples being held at −80°C for 8 days after LN_2_ storage. Each peak corresponds to chemical bonds, and the lack of any significant difference between the pairs implies no major change in alginate structure as a result of a hold at −80°C.

FTIR spectra of the ELS surface kept for 8 days at −80°C (chosen as the time point when significant impairment of ELS had been detected, Figs. 1 and 2) and control beads (thawed rapidly from −196°C) show absorption peaks assignable to calcium alginate at 1600, 1416, 1124, 1090, and 1030 cm^−1^ in Figure 6. Neither shifts in the absorbance peaks nor changes in absorbance ratio relative to the asymmetrical stretching of the carboxylate peak at 1600 cm^−1^ were seen in ELS kept at −80°C for 8 days with respect to the directly thawed control. Shoulders near 1650 and 1550 cm^−1^ correspond to the amide I and amide II of the peptide bonds, respectively, indicating presence of proteins in both ELS sets.

## Discussion

The viable cell number data, Figure 1, show that samples thawed directly from liquid nitrogen recover quickly from cryodamage, which can be explained through cell division and recovery of damage cells. ELS consume glucose during the metabolic process, and these data indicate that a direct thaw from −196°C is better for cell recovery. These glucose results go in line with the observed decrease in viable cell number.

Nonoptimal warming rates can sometimes be blamed for poor cryopreservation outcome. This is not the case in this study–samples held for 1 or 4 h at −80°C experienced the same interruption in cooling rates at −80°C, as those with longer holds showed no additional damage, whereas those with prolonged storage at −80°C on thawing did.

Storing the cells isothermally at −80°C for 8 days is no more damaging than for 1 day at −80°C following liquid nitrogen storage (Fig. 2). Previous work has shown that 1 month storage at −80°C (without the cells experiencing liquid nitrogen temperatures) was damaging to cells (drop in viable cell number of around 30%) and 2 months resulted in a fall in viable cell number of 70% over samples stored below −170°C.^14^ The present data confirm that damage at −80°C increases nonlinearly with time.

The result that short storage at −80°C after liquid nitrogen storage caused severely reduced post-thaw function was somewhat surprising and an effect isolated to the warming phase. The glass transition temperature for a 12% DMSO solution is ∼−120°C.^10,14^ Intracellular material typically has a higher glass transition temperature than the extracellular material consequent of internal proteins, having a higher vitrification temperature,^20^ although its exact value inside DMSO-permeated mammalian cells is unknown.

The damage is possibly related to the fluidizing of the freeze concentrated matrix between the ice crystals on rewarming to −80°C. While ice crystals develop through our entire system, solutes are preferentially excluded from this ice front and eventually vitrify in channels between the ice, and fluidizing of these channels may stress ELS through chemical or osmotic effects resulting in poor post-thaw performance. It is possible that the physical changes that occur when the extracellular material passes through the glass transition point impact the cells resulting in a higher sensitivity to damage at −80°C when the vitrified channels refluidize.

Alternatively, thermal stresses may build in the system due to the large temperature range between −196°C and −80°C. As samples stored for 8 days have much lower survival than those stored for 4 days, and that a 4 h hold at −80°C is not problematic, it is clear that the effect is a longer-term phenomenon, not an instantaneous or very rapid one. Poorer outcomes are not observed with a hold of between 1–4 h on thawing, indicating that the damage is not caused by having a stop in the thermal profile.

The FTIR data show that there is no change in the structure of the alginate component of the ELS that could explain cell death; rather it is a direct effect on the ELS themselves that is in the origin of the damage. While storage at −196°C is preferable to −80°C storage, the data show that damage is not caused by storage only at −80°C or transitions between −80°C and −196°C on the cooling process, rather is an exclusive factor in the warming profile.

Despite its importance to tissue transport, scant research has been published considering challenges of transport and resultant variation of storage temperatures. Publications on samples of arterial and heart valves from the European Homograft Bank state that these tissues cannot be placed back into liquid nitrogen after transfer to −80°C from −196°C, but can be stored at −80°C for 1–3 months after −196°C storage, although damage in this study has been related primarily to thermal-stress cracking.^4,13^

One group has reported that in studying transportation methods with porcine aortic valves, a dry shipper caused maximal damage and that only dry ice transportation was possible. However, this was reportedly due to tissue cracking related to variable warming rates.^7^ ELS monitored in this study do not seem susceptible to major cracking damage, as the liver spheroids are no larger than about 100 μm, and morphology of the ELS is generally well preserved after cryopreservation.^16^ In addition with many grafts, cell survival is not necessary—the scaffold structure is essential and cells can be reseeded later. Warming rates were shown in this study not to be a factor in cell death after −80°C storage.

As more tissue engineered constructs become medically feasible, problems in addition to thermal cracking, such as cell function, will become crucial, and this study helps address those problems. This study shows that the BAL system requires transport at either −196°C or thawed.

Mechanisms have already been developed for short-term ambient temperature storage^21^ and post-vitrification storage while transporting has also been reported with mouse embryos.^22^ However, transporting thawed products can be problematic as recryopreserving the device if not used will prove difficult, and thus, costly products may be wasted.

To summarize, with the current experimental protocols used in this study, ELS should not be transported and/or stored at −80°C after liquid nitrogen storage. Eight-day storage or transport at −80°C after −196°C conditions irreversibly damages the biomass of our BAL device.

## Abbreviations Used

AFP: alpha-1-fetoprotein
BAL: bioartificial liver
DMSO: dimethyl sulfoxide
ELISA: enzyme-linked immunosorbent assay
ELS: encapsulated liver spheroids
FDA: fluorescein diacetate
FTIR: Fourier transform infrared
HBSS: Hanks' buffered saline solution
PI: propidium iodide
RCCS: Rotary Cell Culture System
UW: University of Wisconsin
